# Measuring land surface temperature, near-infrared and short-wave infrared reflectance for estimation of water availability in vegetation

**DOI:** 10.1016/j.mex.2020.101172

**Published:** 2020-12-09

**Authors:** Mauro Holzman, Raúl Rivas, Martín Bayala, José Pasapera

**Affiliations:** a Consejo Nacional de Investigaciones Científicas y Técnicas, Instituto de Hidrología de Llanuras “Dr. Eduardo J. Usunoff” (IHLLA), Rep. Italia 780, B7300 Azul, Argentina; b Comisión de Investigaciones Científicas de la provincia de Buenos Aires, Instituto de Hidrología de Llanuras “Dr. Eduardo J. Usunoff” (IHLLA), Tandil B7000, Argentina; c Comisión Nacional de Investigación y Desarrollo Aeroespacial del Perú-CONIDA, Calle Luis Felipe Villarán 1069, San Isidro, Lima, Perú

**Keywords:** Vegetation water content, Optical/thermal data, Vegetation water stress, Soil moisture, Evapotranspiration

## Abstract

The vegetation water status is a crucial variable for modelling of drought impact, vegetation productivity and water fluxes. Methods for spatial estimation of this variable still need to be improved. The integration of remotely sensed data of land surface temperature (LST) and water vegetation indices based on near-infrared (NIR) and short-wave infrared (SWIR) reflectance for estimation of vegetation water content and water available for evapotranspiration require more analysis. This study contains a detailed method and measurements of LST, NIR and SWIR reflectance of soybean, corn and barley taken in field campaigns in central Argentine Pampas and laboratory with a ST PRO Raytek (8–14 µm) and a spectrometer SVC HR-1024i (0.35 and 2.5 µm). Also, relative water content of leaves was measured in laboratory during the dehydration process. This method and dataset could be also used for researching other wavelengths between 0.35 and 2.5 µm as indicator of water vegetation status (e.g. solar-induced chlorophyll fluorescence, photosynthesis).

• Procedures useful to measure field spectra of vegetation are presented.
• Not only the traditional method to measure leaves spectra in laboratory, but also in field were applied.
• The method allows the integration of spectra and thermal data as a proxy of vegetation water status.

## Graphical abstract

**Figure:**
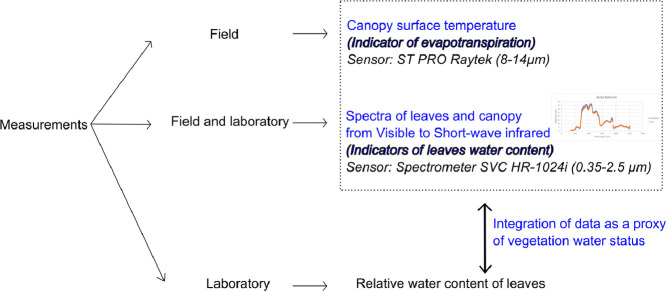

Specification table

**Table:** 

Subject Area	Earth and Planetary Sciences
More specific subject area	*Remote sensing of vegetation water stress*
Method name	*Vegetation water status estimation with optical and thermal data*
Name and reference of original method	
Resource availability	Datasets mentioned in the text are included in https://data.mendeley.com/datasets/5hjyy6z436/1https://digital.cic.gba.gob.ar/handle/11746/10743

## Method details

This article describes the method (and data) applied during three field campaigns over soybean, barley and corn in Argentine Pampas to evaluate the relationship between NIR, SWIR and LST as indicators of water availability in soil-plant system. Reflectance and LST were measured during different campaigns in central-eastern Argentine Pampas (La Campana station, 37°17′ S, 58°56′ W) on soybean, barley and corn rainfed crops. The campaigns were carried out during stages of full vegetation cover and different hydric conditions. Spectra were recorded between 0.35 and 2.50 µm under two condition:

(1) laboratory, on the collected barley and soybean leaves post hydration and during the progressive dehydration process, following previous studies (e.g. [1]) (Table 1, see Supplementary material). Laboratory measurements were taken on 5–7 leaves per plant of soybean and cut plants of barley collected along transects. Leaves of the upper part of the plants were considered, given that they are more representative of the surface monitored by remote sensors. In laboratory, the collected leaves were arranged horizontally over a black surface to avoid the effect of background reflectance, taking spectra on the adaxial surface.

(2) field, every 15 m of each transect over soybean, corn and barley, with which a spectral index of leaf water content based on the difference between NIR (0.850 µm) and SWIR (2.130 µm) reflectance was calculated (Table 2, see Suplementary material). In La Campana station, 2 plots of soybean, 1 of barley and 1 of corn were considered for measuring reflectance along transects during dates of full vegetation cover and different hydric conditions (February 15, 2019 and February 28, 2019 for soybean and corn and during August-December 2019 for barley). Sampling locations were selected such that canopy condition was homogeneous in surrounding areas on scales of 10 m. Measurements were taken under cloud-free conditions and diurnally between 10:30 and 16:00 local time, avoiding significant fluctuations in solar radiation (see meteorological data).

**Fig. 1:**
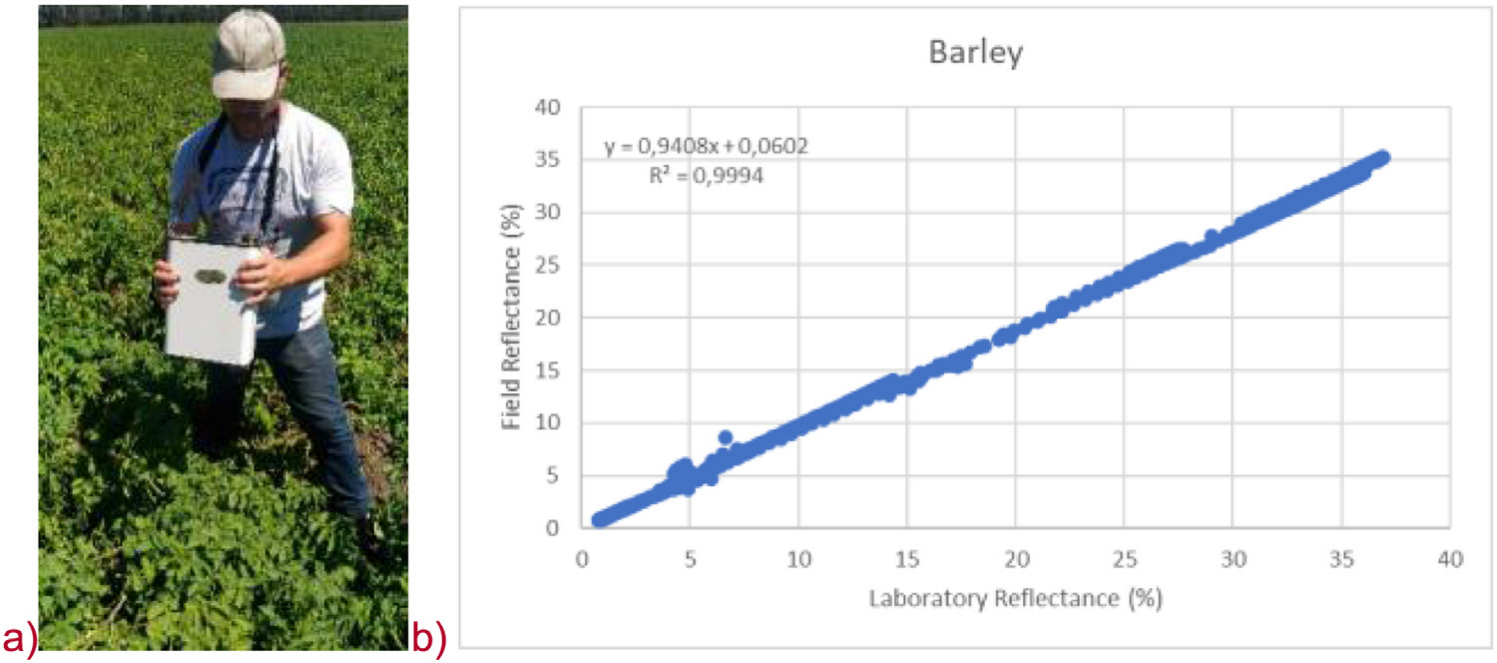
a) Sampling of spectra in field over vegetation with the SVC HR-1024i spectrometer, b) relationship between laboratory and field measured reflectances (0.35–2.50 µm).

Spectra were recorded between 0.35 and 2.50 µm with a spectrometer SVC HR-1024i (Spectra Vista Corporation, USA), FOV=4° This spectrometer can acquire spectra with a sampling interval of ≤ 0.0033 µm, 0.7 µm; ≤ 0.0095 µm, 1.5 µm; ≤ 0.0065 µm, 2.1 µm. A reference reflectance spectralon panel (99%) was used by the spectrometer to convert incident radiance to spectral reflectance. All spectral measurements were made in a nadir orientation, with a distance between the spectrometer and the canopy/leaves about 0.5 m (covering a surface of about 3 cm^2^) to minimize possible effects of shadow of leaves (Fig. 1a). The correlation between laboratory and field reflectance measurements presents a coefficient of determination greater than 0.98 (Fig. 1b).

Foliage temperatures were measured immediately after obtaining spectra with a handheld thermometer ST PRO Raytek (8–14 µm, resolution of 0.1 °C, range 0–50 °C and accuracy about 0.3 °C for the typical range of temperature) (Table 2). LST was obtained hand-holding the thermometer over canopy looking down to the leaves at a distance=0.5 m with an angle of about 70° from the horizontal, that allowed full plant cover. Before taking LST measurements, an Everest black body was used to calibrate the sensor (range of 0–60 °C and accuracy 0.1 °C). Radiometric temperature measurements were corrected for the surface emissivity effect (ε=0.985). LST was measured only in field to consider the effect of water availability on evapotranspiration of crops [2], given that in laboratory the cut leaves/plants cannot show that effect.

The cut samples were immediately weighed in field using a portable electronic balance with an accuracy of 0.01 g (model ES-1200HA, Jadever Scale Co.). After weighing, leaves were placed in sealed plastic bags and transported to laboratory, avoiding drying process. In laboratory, samples were hydrated (≈24 h) with distilled water to determine leaf mass at 100% water content. Afterward, they were exposed to the sun, dried at ambient temperature and then oven-dried at 50 °C until dry weights were constant. Thus, the spectral data of Table 1 are associated to the corresponding relative water content in leaves (RWC,%), a common water content index in plant physiology used to determine plant water status [3]. This is obtained from the difference between actual and dry weight of leaves in relation to the turgid one. The fresh or actual mass was determined during the dehydration process to evaluate the response of spectra to different water content, which was not possible if only hydric condition in field is taken into account. These weights were used to obtain RWC for each sample:

(1) RWC=(Fw−Dw)/(Tw−Dw)100

where Fw is the fresh or actual mass, Dw is the dry mass, Tw is the turgid leaf mass. The difference between fresh and dry mass has been used in previous studies as indicator of vegetation water status [4,5,6,7]. See RWC data in Table 1.

Finally, sensors description, meteorological and energy balance data measured during the campaigns are added in the datasets (see Supplementary material section). Tables 1 and 2 contains the data included in Figs. 5–7 of [4], respectively.

## Declaration of Competing Interest

The authors declare that they have no known competing financial interests or personal relationships that could have appeared to influence the work reported in this paper.

## Appendix Supplementary materials

Image, application 1
